# Quantitative three-dimensional cardiovascular magnetic resonance myocardial perfusion imaging in systole and diastole

**DOI:** 10.1186/1532-429X-16-19

**Published:** 2014-02-24

**Authors:** Manish Motwani, Ananth Kidambi, Steven Sourbron, Timothy A Fairbairn, Akhlaque Uddin, Sebastian Kozerke, John P Greenwood, Sven Plein

**Affiliations:** 1 Multidisciplinary Cardiovascular Research Centre & The Division of Cardiovascular and Diabetes Research, Leeds Institute of Genetics, Health & Therapeutics, University of Leeds, Leeds, UK; 2 Division of Medical Physics, University of Leeds, Leeds, UK; 3 Institute for Biomedical Engineering, University and ETH Zurich, Zurich, Switzerland

**Keywords:** Cardiovascular magnetic resonance, Perfusion, 3-dimensional, Myocardial perfusion imaging, Ischemic heart disease, Myocardial blood flow

## Abstract

**Background:**

Two-dimensional (2D) perfusion cardiovascular magnetic resonance (CMR) remains limited by a lack of complete myocardial coverage. Three-dimensional (3D) perfusion CMR addresses this limitation and has recently been shown to be clinically feasible. However, the feasibility and potential clinical utility of *quantitative* 3D perfusion measurements, as already shown with 2D-perfusion CMR and positron emission tomography, has yet to be evaluated. The influence of systolic or diastolic acquisition on myocardial blood flow (MBF) estimates, diagnostic accuracy and image quality is also unknown for 3D-perfusion CMR. The purpose of this study was to establish the feasibility of quantitative 3D-perfusion CMR for the detection of coronary artery disease (CAD) and to compare systolic and diastolic estimates of MBF.

**Methods:**

Thirty-five patients underwent 3D-perfusion CMR with data acquired at both end-systole and mid-diastole. MBF and myocardial perfusion reserve (MPR) were estimated on a per patient and per territory basis by Fermi-constrained deconvolution. Significant CAD was defined as stenosis ≥70% on quantitative coronary angiography.

**Results:**

Twenty patients had significant CAD (involving 38 out of 105 territories). Stress MBF and MPR had a high diagnostic accuracy for the detection of CAD in both systole (area under curve [AUC]: 0.95 and 0.92, respectively) and diastole (AUC: 0.95 and 0.94). There were no significant differences in the AUCs between systole and diastole (p values >0.05). At stress, diastolic MBF estimates were significantly greater than systolic estimates (no CAD: 3.21 ± 0.50 vs. 2.75 ± 0.42 ml/g/min, p < 0.0001; CAD: 2.13 ± 0.45 vs. 1.98 ± 0.41 ml/g/min, p < 0.0001); but at rest, there were no significant differences (p values >0.05). Image quality was higher in systole than diastole (median score 3 vs. 2, p = 0.002).

**Conclusions:**

Quantitative 3D-perfusion CMR is feasible. Estimates of MBF are significantly different for systole and diastole at stress but diagnostic accuracy to detect CAD is high for both cardiac phases. Better image quality suggests that systolic data acquisition may be preferable.

## Background

Myocardial perfusion imaging with cardiovascular magnetic resonance (CMR) is a highly accurate technique for the detection of coronary artery disease (CAD) [1]. However, conventional acquisition with two-dimensional (2D) methods can only acquire a small number of non-contiguous slices of the left ventricle (LV) at each R-R interval, and therefore incomplete myocardial coverage remains a significant limitation.

Recent technological advances have allowed unprecedented acceleration of dynamic CMR and have led to the development of three-dimensional (3D) myocardial perfusion CMR methods providing full LV coverage with preserved temporal and spatial resolution [2-4]. Three recent studies have shown 3D-perfusion CMR to be clinically feasible and highly accurate for the detection of CAD with visual perfusion assessment [5-7]. However, the feasibility and potential clinical application of deriving quantitative estimates of myocardial blood flow (MBF) from 3D-perfusion CMR has not yet been studied.

A further limitation of conventional 2D-perfusion CMR is that each slice is acquired in a different period of the cardiac cycle. Two recent quantitative studies have shown a significant difference in MBF estimates derived from the same mid-ventricular slice acquired in systole and diastole with 2D-perfusion CMR [8,9]. As well as limiting quantitative comparisons between slices, these significant phasic differences impact on inter-study and longitudinal comparisons of MBF. Unlike 2D-perfusion CMR, 3D imaging allows acquisition of data from the entire myocardium in the same, optimised period of the cardiac cycle. Most previous 3D-perfusion CMR studies have acquired data in systole but to date it is unknown whether systolic or diastolic acquisition leads to better image quality and diagnostic yield. Furthermore, it is unknown whether quantitative estimates of MBF from 3D data demonstrate the same phasic differences previously reported for 2D techniques [8,9].

The purpose of this study was therefore to establish the feasibility of quantitative 3D-perfusion CMR for the detection of coronary artery disease (CAD) and to compare systolic and diastolic estimates of MBF. Defining the optimal cardiac phase for acquisition may be more relevant for 3D than 2D-perfusion CMR because it allows the acquisition of all slices in a particular cardiac phase.

## Methods

### Population

Forty consecutive patients with known or suspected CAD were recruited. All patients were imaged within 30 days of clinically scheduled diagnostic coronary angiography. No revascularization or clinical events occurred between angiography and CMR. Exclusion criteria were contraindications to CMR, adenosine, or gadolinium contrast agents, recent myocardial infarction (MI) or unstable angina (within 6 months), or poorly controlled arrhythmias. Patients were instructed to refrain from caffeine for 24 hours before their CMR study but continue cardiac medications as normal. The study was approved by the regional ethics committee and all patients gave written consent.

### CMR protocol

All studies were performed on a 3.0 T scanner (Achieva TX, Philips Healthcare, Best, The Netherlands) equipped with dual-source parallel radiofrequency transmission technology and a 32-channel cardiac coil. For perfusion imaging, a 3D spoiled turbo gradient-echo sequence was used (TR/TE/flip angle 1.8 ms/0.7 ms/15°; saturation prepulse delay 150 ms; linear *k*-space encoding; 70% partial Fourier acquisition in two dimensions; typical field of view 350 × 350 mm; 10 fold *k*-*t* acceleration and 11 training profiles leading to a net acceleration of 7.0; typical acquisition duration 192 ms, *k*-*t* principal component analysis (PCA) reconstruction; reconstructed to 12 contiguous slices with voxel size 2.3×2.3×5 mm^3^) [2,7].

**Figure 1:**
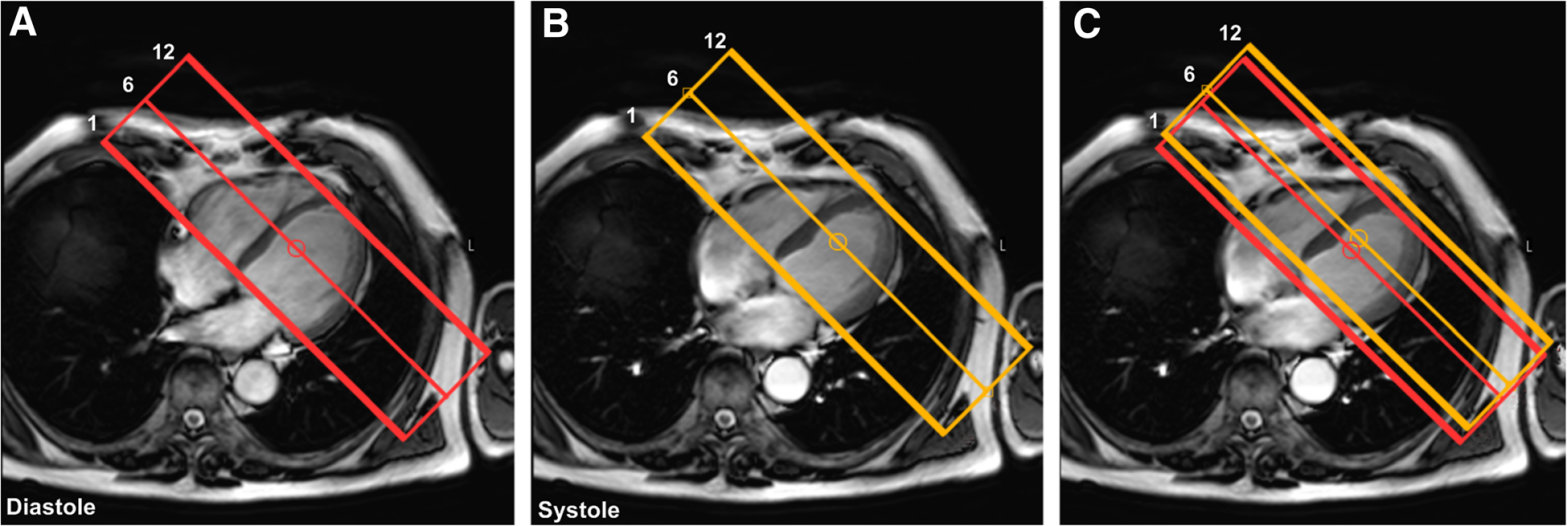
**Acquisition planning.** Because of the longitudinal lengthening of the heart from systole to diastole, the position of the mid-diastolic (red) and end-systolic perfusion stacks (yellow) (12 slices each) were individually planned from the chosen diastolic (Panel **A**) and systolic (Panel **B**) 4-chamber cine frames. Panel **C** shows both stacks superimposed on the chosen end-systolic frame.

Two *k*-*t* undersampled 3D data sets were acquired in each R-R interval, each preceded by a non-selective saturation prepulse. Vertical and horizontal long-axis cine images were used to identify appropriate trigger delays for systolic and diastolic acquisition [8-10]. Additionally, because of the longitudinal lengthening of the heart from systole to diastole, the position of the end-systolic and mid-diastolic perfusion stacks (12 slices each) were individually planned from the chosen systolic and diastolic cine frames (Figure 1) [8,9]. The same trigger delays were used for stress and rest acquisitions.

Stress perfusion images were acquired during intravenous adenosine-induced hyperemia administered for 4 min at 140mcg/kg/min. Consistent with previous 3D-perfusion CMR studies, an intravenous bolus of 0.075 mmol/kg of gadobutrol (Gadovist, Bayer Schering Pharma, Berlin, Germany) was administered at a rate of 4.0 ml/s followed by a 20 ml saline flush. Stress perfusion CMR was followed by cine imaging covering the left ventricle in 10-12 short-axis sections. Rest perfusion CMR was performed 15 min after stress, using identical imaging parameters. Late gadolinium enhancement (LGE) imaging was acquired in the same short-axis geometry as perfusion imaging after an additional 10-15 min using conventional 2D methods (T1 weighted segmented inversion recovery gradient echo; TR/TE/flip angle 4.9 ms/1.9 ms/15°; inversion time individually adjusted according to Look-Locker scan; spatial resolution 1.35 × 1.35 × 10 mm).

### Image quality

Systolic and diastolic perfusion images were analyzed in separate reporting sessions in random order (MM, 2 years experience of perfusion CMR). Overall image quality was scored as follows: 0 = non-diagnostic, 1 = poor, 2 = adequate and 3 = excellent. The occurrence of artifact related to respiratory-motion, *k*-*t* reconstruction or dark-rim artifact was scored as follows: 0 = none, 1 = mild, 2 = moderate and 3 = severe.

### Myocardial blood flow estimation

Perfusion images were processed offline using previously validated in-house software (PMI 0.4; written in IDL 6.4 (ITT Visual Information Systems, Boulder, CO) [11]. All short-axis slices with clearly identifiable LV cavity enhancement during first-pass perfusion and with >75% circumferential LV myocardium were included in the analysis [5,6].

#### Per patient analysis

Following manual rigid motion-correction, a circular region of interest (ROI) was drawn in the basal LV cavity in diastole, to derive the arterial input function (AIF). The same (diastolic) AIF was used for both systolic and diastolic estimates of MBF in order to avoid potential variations in the AIF between phases with subsequent effects on MBF estimation [8].

A whole-heart myocardial region of interest (ROI) excluding any dark-rim artifact was drawn for both systolic and diastolic perfusion images (covering all slices containing myocardium). Signal intensity–time data were converted to concentration-time data by subtracting the baseline signal, and global MBF was estimated at stress and rest using constrained deconvolution with a delayed Fermi-model applied to the first pass [9,12,13]. MPR was calculated as stress MBF divided by rest MBF.

### Per territory analysis

The above analysis was repeated on a per territory basis using the 17-segment AHA model adjusted for coronary dominance [14]. For this, all slices from an individual patient were first visually allocated to basal, mid or apical sections of the model. For each perfusion territory, a myocardial ROI was then outlined including all segments pertaining to that territory across all slices according to the 17-segment AHA model. MBF and MPR estimates were obtained using the same algorithm as for the whole-heart ROI.

### Intra-observer and inter-observer variability

Thirty random territories were re-analyzed 1 month later by the same observer (M.M.) and by a second observer A.K. (2 yrs and 1 yr experience respectively). A.K. was blinded to the results of all previous analyses.

### Quantitative coronary angiography

Quantitative coronary angiography was performed (QCAPlus, Sanders Data Systems, Palo Alto, CA, USA) on anonymised X-ray angiography images (M.M. 6 years experience in coronary angiography). Significant CAD was defined as luminal stenosis ≥70% diameter in any of the main epicardial coronary arteries or their branches with a diameter of ≥2 mm.

### Statistical analysis

Analysis was performed using SPSS 17.0 (SPSS, Chicago, IL). Data are presented as mean ± SD. Group means were compared using paired or unpaired Student t-tests; or within-subjects analysis of variance with Greenhouse-Geisser correction for multi-sample sphericity, as appropriate. Ordinal data were compared using the Wilcoxon signed-rank test. Receiver-operating characteristic (ROC) analysis was performed on a per territory basis, to determine the diagnostic accuracy of MPR to detect significant CAD. Diagnostic accuracies are presented as area under the ROC curve (AUC); and were compared between systole and diastole using methods described by Delong and Delong. Optimal MPR cut-off values, for both cardiac phases, were defined as values that maximised the sum of sensitivity and specificity. A secondary ROC analysis was performed to evaluate the diagnostic accuracy of stress MBF diastolic/systolic ratio. To assess reproducibility, the coefficients of variation (CoV) for intra- and inter-observer measurements were calculated. Because three coronary territories were examined per patient, the intra-cluster correlation coefficient (ICC) was calculated for MPR estimates to determine the design effect and the need to adjust data for clustering. All statistical tests were 2-tailed and a p value <0.05 was considered significant.

## Results

### Study population

**Figure 2:**
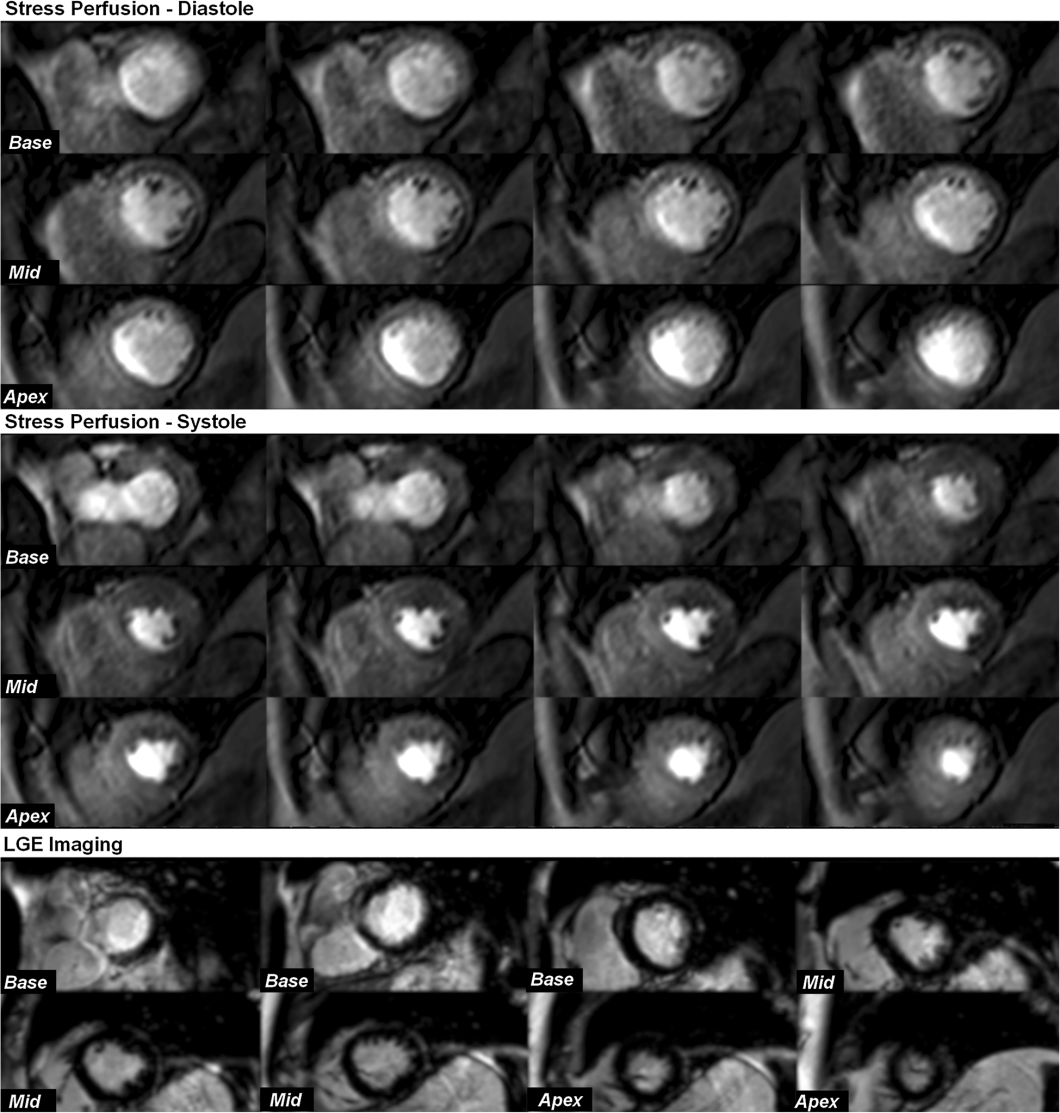
**Case example: 3D-perfusion CMR in systole and diastole.** This example shows 3D-perfusion CMR in a 75-year-old man with angina. Stress-induced perfusion defects are seen infero-laterally from base to apex and antero-laterally from mid-ventricle to apex in both diastole and systole. However, perfusion defects are difficult to discern from dark-rim artifact in diastole and are more clearly delineated with systolic acquisition. Late-gadolinium enhancement imaging did not reveal any myocardial infarction. X-ray coronary angiography revealed 80% stenosis of a large diagonal branch and significant proximal disease in a large dominant left circumflex artery.

Five of the 40 recruited patients were excluded: 3 because of claustrophobia and 2 owing to technical problems (1 mistimed contrast injection; 1 significantly altered patient position between stress and rest scans). A total of 35 patients (105 coronary territories) were therefore available for analysis. Table 1 shows the baseline patient characteristics. QCA confirmed significant CAD in 20 patients (57%) and 38 coronary territories (36%). Only 3 patients had evidence of MI on LGE imaging (the same 3 patients with a clinical history of MI), and this involved only 3 of the 105 territories analysed. Figure 2 shows an example of the stress perfusion images acquired in a patient with significant CAD.

**Table 1:** Patient characteristics

***Parameter***	***Data (n = 35)***
**Age(yrs ± SD)**	62 ± 8
**Sex, n (%)**	
Male	26 (74)
Female	9 (26)
**Risk factors, n (%)**	
Hypertension	18 (51)
Hypercholesterolemia	19 (54)
Diabetes Mellitus	6 (17)
Smoking	14 (40)
Previous MI	3 (9)
Previous PCI	3 (9)
Angiography findings,n (%) *	
No significant disease	15 (43)
One-vessel disease	6 (17)
Two-vessel disease	10 (29)
Three -vessel disease	4 (11)
LAD disease	17 (49)
LCX disease	10 (29)
RCA disease	11 (31)

*Significant disease defined as coronary stenosis ≥70% on quantitative coronary analysis.

*MI* = myocardial infarction; *PCI* = percutaneous coronary intervention; *LAD* = left anterior descending coronary artery; *LCX* = left circumflex coronary artery; *RCA* = right coronary artery.

### Image quality

Overall image quality was better in systole than in diastole (median image quality score: 3 vs. 2 respectively; p = 0.002). In diastole, there was a greater frequency of dark-rim artifact (19 patients [54%] vs. 9 patients [26%] and a higher overall artifact score compared to systole (median scores: 1 vs. 0 respectively; p < 0.0001).

In 5 patients (14%), perfusion images (both cardiac phases) were affected by *k*-*t* reconstruction artifacts at stress and/or rest due to respiratory motion, but all of these artifacts occurred at the end of the breath-hold and did not affect analysis of the first-pass perfusion images.

### Myocardial blood flow estimation

Estimates of MBF and MPR for both cardiac phases are seen in Tables 2, 3 and 4. On per patient (n = 35) and per territory analysis (n = 105), mean resting MBF was similar in both cardiac phases (all p values > 0.05); but mean stress MBF and MPR were significantly greater in diastole than systole (all p values <0.001). These relationships existed in normal and CAD subgroups, as well as overall (all p values <0.01) (Tables 2, 3, 4). In both cardiac phases, stress MBF and MPR were significantly lower in the presence of CAD than in normal patients (all p values < 0.01) or normal territories (all p values < 0.0001) (Tables 2, 3, 4).

**Table 2:** Estimates of MBF and MPR from 3D perfusion CMR - per patient analysis

	**Stress MBF (ml/min/g)**			**Rest MBF (ml/min/g)**			**MPR**		
	***Systole***	***Diastole***	***p***	***Systole***	***Diastole***	***p***	***Systole***	***Diastole***	***p***
***Normal (n = 15)***	2.88 ± 0.32	3.47 ± 0.41	p < 0.0001	1.28 ± 0.17	1.26 ± 0.15	p = 0.45	2.27 ± 0.37	2.78 ± 0.40	p < 0.0001
***CAD (n = 20)***	2.32 ± 0.42	2.53 ± 0.47	p < 0.0001	1.32 ± 0.19	1.28 ± 0.21	p = 0.06	1.82 ± 0.54	2.08 ± 0.74	p < 0.001
***Overall (n = 35)***	2.56 ± 0.47	2.93 ± 0.65	p < 0.0001	1.30 ± 0.18	1.27 ± 0.19	p = 0.06	2.01 ± 0.08	2.38 ± 0.05	p < 0.0001

All values expressed as mean ± SD. Coronary artery disease (*CAD*) defined as stenosis ≥70%. *CMR* = cardiovascular magnetic resonance; *MBF* = myocardial blood flow; *MPR* = myocardial perfusion reserve.

**Table 3:** Estimates of MBF and MPR from 3D-perfusion CMR - per territory analysis

	**Stress MBF (ml/min/g)**			**Rest MBF (ml/min/g)**			**MPR**		
	***Systole***	***Diastole***	***p***	***Systole***	***Diastole***	***p***	***Systole***	***Diastole***	***p***
***Normal (n=*** ***67)***	2.75 ± 0.42	3.21 ± 0.50	p < 0.0001	1.24 ± 0.15	1.25 ± 0.15	p = 0.27	2.26 ± 0.43	2.59 ± 0.44	p < 0.0001
***CAD (n=*** ***38)***	1.98 ± 0.41	2.13 ± 0.55	p < 0.0001	1.24 ± 0.15	1.26 ± 0.14	p = 0.20	1.63 ± 0.14	1.72 ± 0.19	p < 0.01
***Overall (n=*** ***105)***	2.47 ± 0.55	2.82 ± 0.71	p < 0.0001	1.24 ± 0.15	1.25 ± 0.15	p = 0.10	2.03 ± 0.52	2.27 ± 0.61	p < 0.0001

All values expressed as mean ± SD. Coronary artery disease (*CAD*) defined as stenosis ≥70%. *CMR* = cardiovascular magnetic resonance; *MBF* = myocardial blood flow; *MPR* = myocardial perfusion reserve.

**Table 4:** **Regional estimates of MBF and MPR** - **per territory** (**n**=**105)**

	**Normal**			**CAD**		
	***Systole***	***Diastole***	***p***	***Systole***	***Diastole***	***p***
**Stress MBF (ml/min/g)**						
*LAD*	2.91 ± 0.35	3.43 ± 0.46	p<0.0001	2.11 ± 0.46	2.28 ± 0.52	p<0.001
*LCX*	2.63 ± 0.33	3.10 ± 0.44	p<0.0001	1.90 ± 0.28	2.04 ± 0.28	p<0.01
*RCA*	2.77 ± 0.51	3.16 ± 0.55	p<0.0001	1.85 ± 0.39	1.97 ± 0.40	p<0.0001
**Rest MBF (ml/min/g)**						
* LAD*	1.26 ± 0.17	1.30 ± 0.17	p=0.10	1.21 ± 0.12	1.23 ± 0.12	p=0.52
* LCX*	1.26 ± 0.17	1.24 ± 0.13	p=0.56	1.22 ± 0.14	1.20 ± 0.10	p=0.27
* RCA*	1.20 ± 0.13	1.23 ± 0.15	p=0.28	1.28 ± 0.13	1.24 ± 0.16	p=0.26
**MPR**						
* LAD*	2.35 ± 0.42	2.68 ± 0.48	p<0.0001	1.75 ± 0.37	1.87 ± 0.51	p=0.02
* LCX*	2.12 ± 0.35	2.52 ± 0.38	p<0.0001	1.58 ± 0.32	1.72 ± 0.29	p<0.01
* RCA*	2.33 ± 0.51	2.59 ± 0.48	p<0.001	1.46 ± 0.34	1.60 ± 0.34	p<0.01

All values expressed as mean ± SD. Coronary artery disease (*CAD*) defined as stenosis ≥70%. *MBF* = myocardial blood flow; *MPR* = myocardial perfusion reserve; *LAD* = left anterior descending coronary artery; *LCX* = left circumflex coronary artery; *RCA*= right coronary artery.

Analysis of the normal patient group (n = 15) found no significant regional differences in stress MBF, rest MBF or MPR between the LAD, LCX or RCA perfusion territories in both phases (all p values <0.05) (Table 5).

**Table 5:** **Comparison of regional MBF and MPR estimates** – **in normal patients** (**n**=**15**)

	**Perfusion territory**			
	***LAD***	***LCX***	***RCA***	***p***
**Stress MBF (ml/min/g)**				
Systole	2.95 ± 0.36	2.66 ± 0.29	2.88 ± 0.46	P=0.11
Diastole	3.51 ± 0.46	3.19 ± 0.27	3.27 ± 0.47	P=0.10
**Rest MBF (ml/min/g)**				
Systole	1.27 ± 0.17	1.29 ± 0.19	1.20 ± 0.13	p=0.25
Diastole	1.29 ± 0.17	1.27 ± 0.14	1.22 ± 0.15	p=0.47
**MPR**				
Systole	2.36 ± 0.42	2.10 ± 0.32	2.45 ± 0.53	P=0.06
Diastole	2.76 ± 0.45	2.54 ± 0.33	2.71 ± 0.48	P=0.27

All values expressed as mean ± SD. *MBF* = myocardial blood flow; *MPR* = myocardial perfusion reserve; *LAD* = left anterior descending coronary artery; *LCX* = left circumflex coronary artery; *RCA*= right coronary artery.

Based on MPR estimates, the ICC for this study was low (r1 =0.09 [95% CI: -0.25 to 0.41]) with a design effect of 1.18 where cluster size = 3. This shows that the study design of using 3 ‘related’ coronary territories per patient (n = 35) to derive a sample size of 105 coronary territories does not significantly diminish statistical power.

### Diagnostic performance

#### Stress MBF

**Figure 3:**
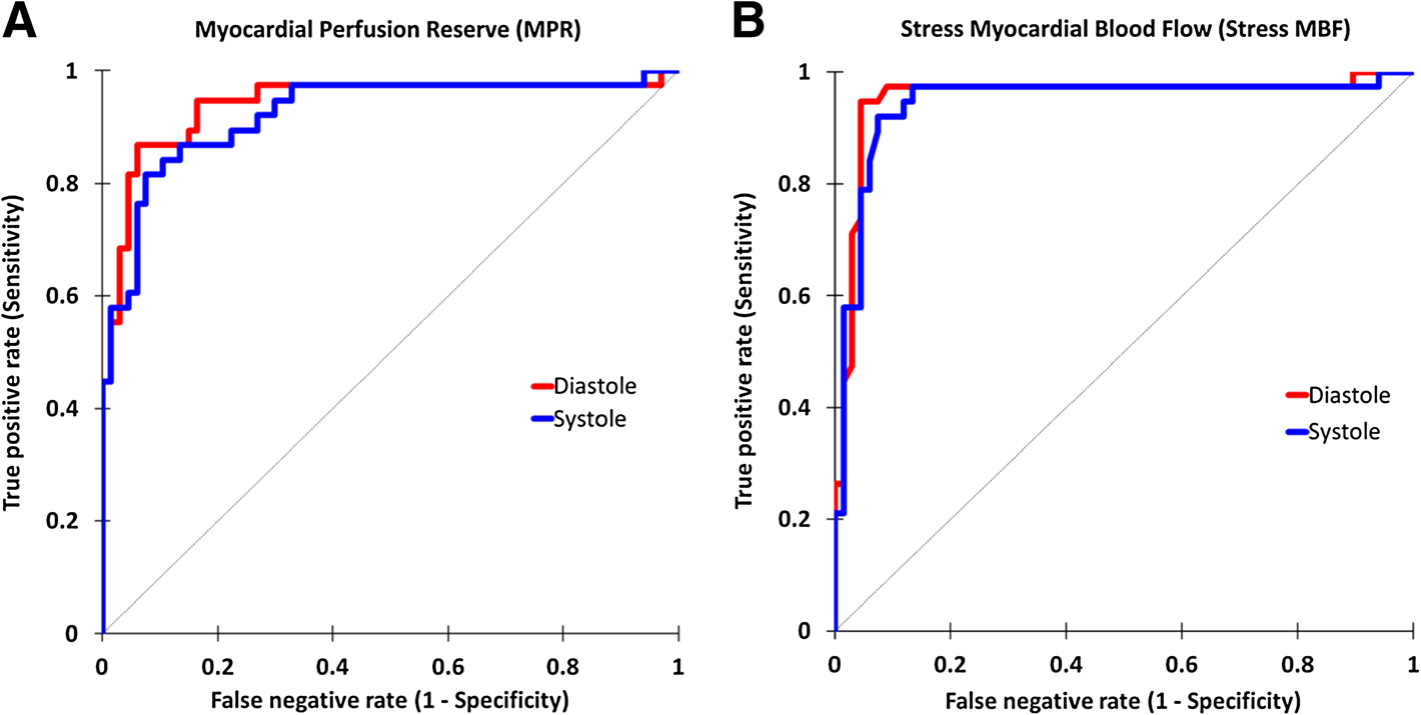
**Diagnostic accuracy of quantitative 3D-perfusion CMR. (A)** Receiver-operator characteristic curves shows a high diagnostic accuracy in both systole and diastole for MPR (area under curve [AUC]: 0.92 vs. 0.94 respectively; p = 0.41). **(B)** Use of stress MBF alone also had a high diagnostic accuracy in both cardiac phases (AUC: 0.95 for both; p = 0.70) and in fact there was no significant difference compared to MPR (p > 0.05 for both cardiac phases).

On a per territory analysis (n = 105), the use of stress MBF alone had a high overall diagnostic accuracy for the detection of CAD - which was similar in both cardiac phases (AUC = 0.95 for both; p = 0.70). The optimal stress MBF cut-off value was 2.31 ml/min/g for systole and 2.60 ml/min/g for diastole. At these thresholds, the sensitivity and specificity were 92% and 93% respectively for systole; and 95% and 96% for diastole. There was no significant difference between the diagnostic accuracy of MPR or stress MBF alone for either cardiac phase (both p values >0.05) (Figure 3).

### MPR

**Figure 4:**
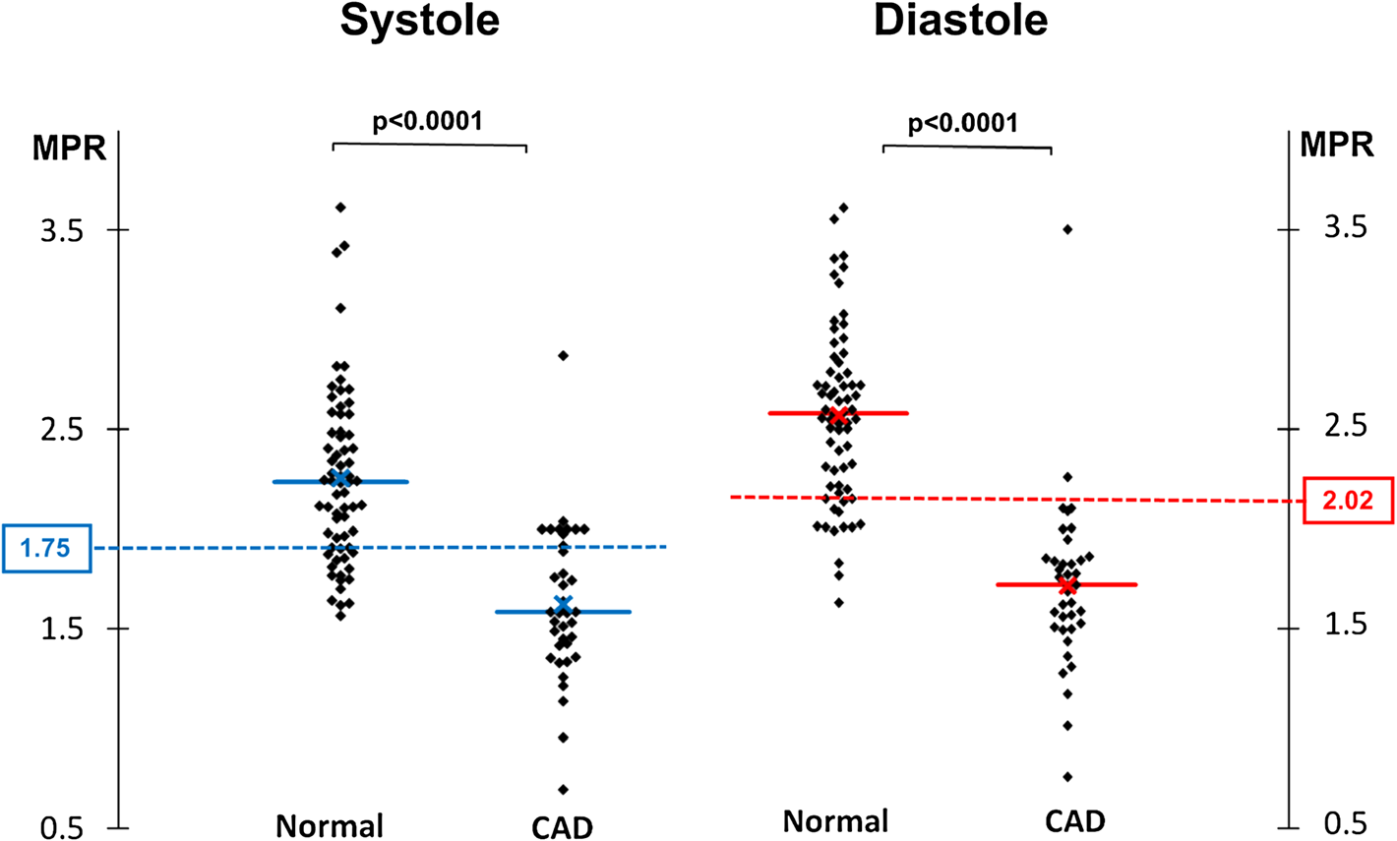
**Myocardial perfusion reserve threshold.** The scatter-plots show myocardial perfusion reserve (MPR) values from normal and significantly diseased perfusion territories with both systolic and diastolic 3D-perfusion CMR (x = mean value, solid line = median value). The optimal MPR cut-off values determined by receiver-operating characteristic analysis are also plotted (dashed lines, 1.75 for systole and 2.02 for diastole).

On per territory analysis (n = 105), MPR also had a high overall diagnostic accuracy for the detection of significant CAD, and this was similar in both cardiac phases (AUC, systole: 0.92 vs. diastole: 0.94; p = 0.41) (Figure 3). The optimal MPR cut-off value was 1.75 for systole and 2.02 for diastole (Figure 4). At these thresholds, the sensitivity and specificity were 82% and 93% respectively for systole; and 87% and 94% for diastole. The diagnostic accuracy of MPR to detect CAD in each of the 3 coronary territories is shown in Table 6 and no significant differences were seen between cardiac phases.

**Table 6:** **Diagnostic accuracy of quantitative 3D**-p**erfusion CMR**–**per territory** (**n**=**105**)

	**AUC for MPR**		
	***Systole***	***Diastole***	***P***
***All territories***	0.92 (0.87-0.98)	0.94 (0.88-0.99)	p=0.41
***LAD***	0.89 (0.78-0.99)	0.90 (0.79-1.00)	p=0.76
***LCX***	0.88 (0.77-0.99)	0.98 (0.93-1.00)	p=0.34
***RCA***	0.92 (0.86-0.99)	0.98 (0.93-1.00)	p=0.50

Values are area under the curve (*AUC*) and (95% confidence interval). *LAD* = left anterior descending coronary artery; *LCX* = left circumflex coronary artery; *RCA*= right coronary artery.

### Diastolic/systolic stress MBF ratio

**Figure 5:**
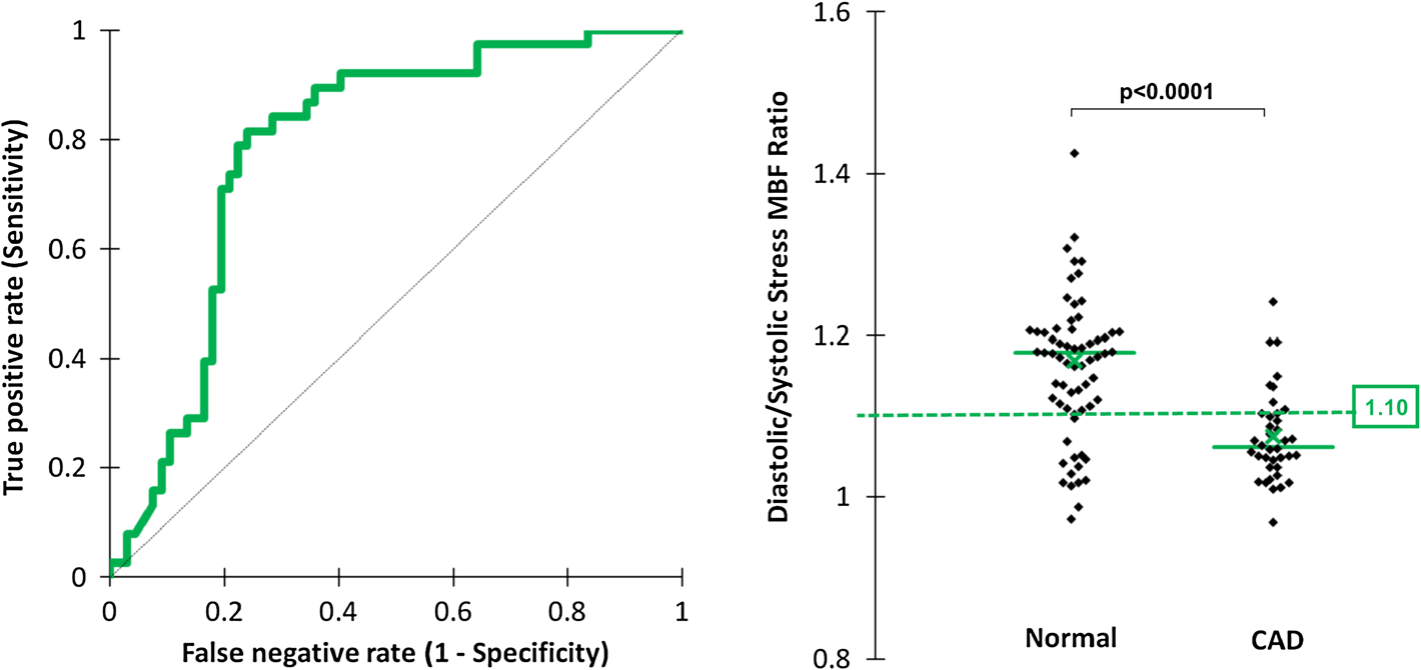
**Diastolic/systolic stress myocardial blood flow ratio.** The ratio of diastolic to systolic myocardial blood flow at stress was significantly lower for territories with coronary artery disease (CAD) than in normal territories (1.07 ± 0.06 vs. 1.17 ± 0.11; p < 0.0001). On receiver-operator characteristic analysis, the diagnostic accuracy (area under the curve) of this ratio to detect significant CAD was 0.79. The optimal cut-off value for the ratio was 1.10, which gave a sensitivity of 82% and specificity of 76%.

The diastolic/systolic stress MBF ratio was significantly lower for territories with CAD than in normal territories (1.07 ± 0.06 vs. 1.17 ± 0.11; p < 0.0001). On ROC analysis, the diagnostic accuracy (AUC) of this ratio to detect significant CAD was 0.79. The optimal cut-off value for the ratio was 1.10 which gave a sensitivity of 82% and specificity of 76% (Figure 5).

### Reproducibility

#### Stress MBF

The mean absolute difference between intra-observer measurements of stress MBF was similar in systole and diastole (0.33 ± 0.14 vs. 0.35 ± 0.16; p = 0.18); and the corresponding CoVs were 16% and 17% respectively. The mean absolute difference between inter-observer measurements of stress MBF was also similar in systole and diastole (0.41 ± 0.22 vs. 0.45 ± 0.20; p = 0.11) with corresponding CoVs of 18% for both.

### MPR

The mean absolute difference between intra-observer measurements of MPR was similar in systole and diastole (0.30 ± 0.15 vs. 0.36 ± 0.13; p = 0.09); and the corresponding CoVs were both 18%. The mean absolute difference between inter-observer measurements of MPR was also similar in systole and diastole (0.35 ± 0.17 vs. 0.41 ± 0.15; p = 0.07) with corresponding CoVs of 20% and 21% respectively.

## Discussion

The main findings of this study are 1) quantitative 3D-perfusion CMR is feasible and has a high diagnostic accuracy for the detection of CAD; 2) similar to 2D studies, estimates of stress MBF and MPR from 3D data are significantly greater in diastole than systole; and 3) the diastolic dominance of stress MBF estimates is reduced in ischemia.

One of the limitations of myocardial perfusion imaging and standard visual interpretation is the dependence on a reference area of normal perfusion. This is a particular impediment in diffuse or balanced multi-vessel disease. This limitation can be avoided by using absolute quantification of MBF [15]. At present, the most robust technique to quantify MBF noninvasively is positron emission tomography (PET) - but its wide-spread clinical application has been slowed by limited access [16]. PET imaging also involves exposure to ionizing radiation, and its spatial resolution limits evaluation of transmural flow differences in normal thickness myocardium.

Over the last decade, several animal, normal volunteer and patient studies have validated the use of CMR for absolute MBF quantification against microsphere and invasive coronary flow reserve measurements [17-19]. Furthermore, several clinical studies have demonstrated high diagnostic accuracy of CMR derived estimates of absolute MBF and MPR against both QCA and fractional flow reserve [9,13,15,20,21]. Nonetheless, the lack of complete myocardial coverage has been a significant limitation of conventional 2D-perfusion CMR for this purpose.

This study has for the first time demonstrated the feasibility of quantitative whole-heart 3D-perfusion CMR. Shin *et al* have previously reported semi-quantitative measures (time-intensity curve indices) of resting myocardial perfusion from a 3D acquisition in 3 healthy volunteers - but no stress acquisition or absolute MBF quantification was performed [10]. The MBF values derived with 3D-perfusion CMR in normal patients in the present study are comparable to values from PET studies and the previous CMR literature [22,23]. For example, in a large study of 160 healthy men and women with PET, the mean resting MBF was 0.98 ± 0.23 ml/min/g (range 0.59-2.05 ml/min/g) and the mean stress MBF was 3.77 ± 0.85 ml/min/g (range 1.85-5.99 ml/min/g) [23]. Intra- and inter-observer reproducibility for stress MBF and MPR in our study was also similar to that seen in 2D-perfusion CMR and PET studies [24,25].

The finding of lower estimates of stress MBF in systole compared to diastole is consistent with the expected physiology and a number of previous studies. Physiologically, one explanation is that during systole, the effect of adenosine-mediated vasodilatation is diminished by the compression of intramyocardial vessels [26]. Two previous 2D-perfusion CMR studies have shown the same phasic differences with higher stress MBF estimates in diastole, but no difference between the phases at rest [8,9]. One previous 3D-perfusion CMR study confirmed similar semi-quantitative measures of resting myocardial perfusion between systole and diastole - but no stress perfusion was performed [10]. Our study has now demonstrated that these phasic differences are also seen with 3D-perfusion CMR quantification and underline the importance of stating the phase of acquisition in future studies to allow comparison in the literature.

Quantitative analysis with MPR yielded high diagnostic accuracies in both systole and diastole (AUC: 0.92 and 0.94 respectively). The optimal MPR cut-off values for detecting significant CAD (1.75 for systole and 2.02 for diastole) were within the range of 1.50–2.06 reported in previous 2D-perfusion CMR studies [9,13,20,21,27]. Recently, in 2D-perfusion CMR, Huber *et al* (n = 31) showed that the use of stress MBF alone had a similar diagnostic accuracy as MPR (AUC 0.92 vs. 0.84 respectively; p < 0.18) [11]. Our study has shown a similar finding in 3D-perfusion CMR and the implication is that a rest perfusion sequence could potentially be omitted in quantitative studies, thus reducing both scanning and post-processing times without a loss in diagnostic yield (Figure 3).

The noted reduction in diastolic/systolic stress MBF ratio in territories with CAD is consistent with previous invasive studies measuring coronary flow velocity throughout the cardiac cycle [28]. The loss of diastolic dominance has been explained by the increased influence of a significant stenosis on flow during periods of low vascular resistance and low intramyocardial tension (diastole); as compared with that during periods of high vascular resistance and high intramyocardial tension (systole) [29]. As such, the diastolic/systolic stress MBF ratio is a novel diagnostic index with moderate diagnostic accuracy (AUC = 0.79) – and this may warrant further evaluation in future studies.

In our quantitative study, both phases had similar diagnostic performance and reproducibility. However, similar to previous 2D studies, diastole was more prone to dark-rim artifact with an adverse effect on image quality; and this is thought to relate to the thinner myocardium, making it more prone to partial volume effects at a given spatial resolution [9]. For this reason, as well as the fact that contour delineation is easier in systole because of the thicker myocardium, we would suggest systole as the preferred phase for 3D-perfusion CMR acquisition - particularly for quantitative studies. Although analysis time was not specifically measured, each 3D perfusion dataset took approximately 20 min to analyse on a per patient basis (including stress and rest analyses for either the systolic or diastolic cardiac phase). Quantifying 3D-perfusion CMR can be simpler than quantifying conventional 2D datasets, because as in our study, fewer dynamic images are often acquired and there is a degree of temporal filtering due to the undersampling in the temporal domain which reduces the amount of time-consuming manual motion correction required.

Finally, there is considerable scope for quantitative perfusion CMR in clinical practice and therefore demonstrating the feasibility of 3D whole-heart coverage and quantification is important. Nonetheless, there still remain a number of other limitations that hold back the wider clinical adoption of quantitative perfusion CMR. The current lack of standardisation in image acquisition, contrast dosing protocols, post-processing, mathematical modelling and interpretation is addressed by an international standardisation task force [30,31]. There is also no widely available and validated analysis software for quantitative analysis of perfusion CMR data and research groups generally use in-house solutions. Analysis can be time-consuming, precluding routine clinical application. Finally, the incremental value of quantitative analysis of myocardial perfusion CMR analysis needs to be shown in large clinical studies.

### Study limitations

The spatio-temporal undersampling methods required for 3D data acquisition are sensitive to respiratory motion, cardiac arrhythmia and low-pass temporal filtering - all of which pose additional challenges to quantitative assessment. Low-pass temporal filtering in particular may lead to underestimation of MBF. We reduced these limitations by use of the constrained *k*-*t* PCA framework for image reconstruction, which has been shown to improve temporal fidelity, permitting robust measurements of MBF at very high acceleration factors [32]. The latter is also less prone to respiratory artifact as temporal basis functions are derived based on the low-resolution training data acquired in every heartbeat [2].

Although MPR performed well in our study, perfusion imaging is a measurement of the hemodynamic consequences of a stenosis rather than its anatomy, and therefore our use of QCA is an imperfect reference standard. Following this initial feasibility study, future validation against fractional flow reserve is planned in a larger clinical population. Another limitation, common to many previous studies, is the potential effect of data clustering as three perfusion territories are examined per patient [20,27]. However, the design effect of this was low (1.18) owing to a small ICC and cluster size.

Finally, the model used for estimating MBF assumes a linear relation between signal and contrast agent concentration i.e. ignoring saturation effects in the LV blood pool which can lead to underestimation of MBF [33]. This is particularly relevant as we used a relatively high contrast agent dose of 0.075 mmol/kg bodyweight to be consistent with previous 3D-perfusion CMR studies. Proposed solutions include the use of a non-linear signal model combined with precontrast T1-mapping and/or the use of a small pre-bolus to measure the AIF. There is currently no evidence that either of these potential solutions actually leads to improved diagnostic accuracy for the detection of CAD in the clinical setting. In fact the only study directly addressing this question came to the opposite conclusion i.e. the use of a pre-bolus AIF was found to reduce diagnostic accuracy compared to a single-bolus approach [34].

## Conclusions

We have shown that quantitative 3D-perfusion CMR is feasible and can be used to detect CAD with high diagnostic accuracy. In addition, we have found that there are significant differences in systolic and diastolic MBF estimates. Both cardiac phases provide comparable diagnostic yield, albeit at different thresholds. Because systolic images had fewer artifacts and higher image quality, systole may be the preferred phase for acquisition of 3D perfusion data.

## Abbreviations

CAD: Coronary artery disease; CMR: Cardiovascular magnetic resonance; MBF: Myocardial blood flow; MPR: Myocardial perfusion reserve; k-t: *k*-space and time; PCA: Principal component analysis; ROI: Region of interest; AIF: Arterial input function; AUC: Area-under-the-curve; ROC: Receiver-operator characteristic.

## Competing interests

The authors declare that they have no competing interests. SP and AK are funded by a British Heart Foundation Senior Clinical Research Fellowship (FS/1062/28409). SP and JPG receive an educational research grant from Philips Healthcare.

## Authors’ contributions

MM: conception and design, acquisition, analysis and interpretation of data, drafting of manuscript; AK: acquisition, analysis and interpretation of data, critical and intellectual revision of manuscript; SS: analysis and interpretation of data, critical and intellectual revision of manuscript; TAF: critical and intellectual revision of manuscript; AU: acquisition of data, critical and intellectual revision of manuscript; SK: critical and intellectual revision of manuscript; JPG: interpretation of data, critical and intellectual revision of manuscript; SP: conception and design, interpretation of data, critical and intellectual revision of manuscript. All authors read and approved the final manuscript.
